# Can Centralized Sanctioning Promote Trust in Social Dilemmas? A Two-Level Trust Game with Incomplete Information

**DOI:** 10.1371/journal.pone.0124513

**Published:** 2015-04-16

**Authors:** Raymond Yu Wang, Cho Nam Ng

**Affiliations:** 1 Faculty of Social Sciences, The University of Hong Kong, Pokfulam, Hong Kong, Hong Kong; 2 Department of Geography, The University of Hong Kong, Pokfulam, Hong Kong, Hong Kong; George Mason University, UNITED STATES

## Abstract

The problem of trust is a paradigmatic social dilemma. Previous literature has paid much academic attention on effects of peer punishment and altruistic third-party punishment on trust and human cooperation in dyadic interactions. However, the effects of centralized sanctioning institutions on decentralized reciprocity in hierarchical interactions remain to be further explored. This paper presents a formal two-level trust game with incomplete information which adds an authority as a strategic purposive actor into the traditional trust game. This model allows scholars to examine the problem of trust in more complex game theoretic configurations. The analysis demonstrates how the centralized institutions might change the dynamics of reciprocity between the trustor and the trustee. Findings suggest that the sequential equilibria of the newly proposed two-level model simultaneously include the risk of placing trust for the trustor and the temptation of short-term defection for the trustee. Moreover, they have shown that even a slight uncertainty about the type of the newly introduced authority might facilitate the establishment of trust and reciprocity in social dilemmas.

## Introduction

**Fig 1:**
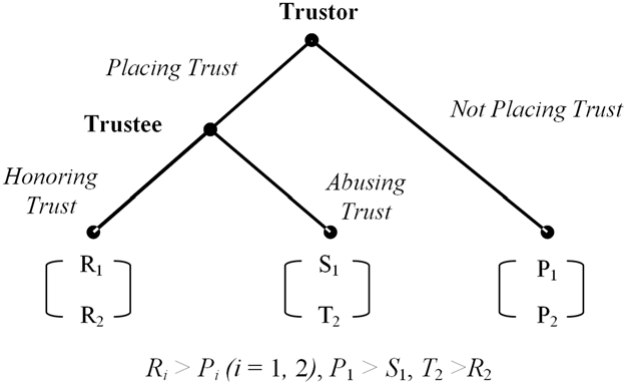
Extensive form of the Trust Game.

Trust is a critical social factor which is considered to be highly conducive to preventing opportunistic behaviour, decreasing transaction costs and maintaining cooperation in human activities [1–5]. Traditional game theoretic analysis of trust is usually built upon a standard trust game as shown in Fig 1 [6–11]. In the trust game, “not placing trust” is the rational individual action in a one-shot game [12]. This is a suboptimal social outcome because “placing trust, honouring trust” is a strict improvement for both the trustor and the trustee. From this point of view, Fig 1 concisely illustrates a paradigmatic social dilemma in human society, where *R*_1_, *S*_1_, *P*_1_ denote the utility of the trustor and *R*_2_, *T*_2_, *P*_2_ denote the utility of the trustee.

However, the traditional trust game has limitations of analysing hierarchical interactions in complex systems. In particular, what is missing in the traditional model is a dynamic perspective which distinguishes different types of trust. In many conventional studies, trust is a concept at the individual or interpersonal level [13]. Some scholars focus on interpretations of one single type of trust such as inter-personal relationships in which trust is a stationary result under certain socio-economic conditions [14–23]. Others highlight the sources of trust and trace them back to the process of interactions or characteristics and competence of individuals [24–26]. Yet if one analyses the problem of trust in more complex contexts, then it is important to recognize that it involves interactions between more than just individuals [27]. Consider common-pool resource (CPR) governance in a large-scale social-ecological system (SES), it is evident that trust should be attributed to a variety of relationships between multiple groups, organizations and institutions. Under diverse social, economic, political and spatial-temporal conditions, noticeably, an actor has to measure his belief not only in the trustworthiness of his peer actors, but also in institutions and governance agencies which influence collective and individual behaviour through external intervention. These trust relationships are beyond social-psychological understandings of inter-personal activities. Against the backdrop of increasing complexity, the problem of trust thus should be examined through an alternative structure which integrates interactions between actors at different levels.

From the perspective of rational choice theory, formal models and experiments have been developed to investigate dynamics and mechanisms of trust relationships [10,28,29]. Built on dyadic interactions between different actors, these theoretical and experimental studies have shown that reciprocity and peer punishment can facilitate trust in social dilemmas [12,30,31]. However, the major limitation of these models is that they are established on a horizontal actor-network in which only individual trust is examined. One should notice that peer punishment on defectors through reciprocity is difficult in more complicated systems [32,33]. Recently, scholars have started raising questions in regard to “the ability of spontaneous, uncoordinated and decentralized peer punishment to sustain cooperation in complex societies”. Based on experiments of public good games (PGG), some scholars argue that peer punishment might be only effective under conditions such that the group size is sufficiently small or exit option is provided to participants [34–37]. In empirical settings of CPR governance, we frequently observe centralized institutions which impose top-down sanctions to prevent opportunistic behaviour. Some experimental evidence derived from PGG also has shown that centralized sanctioning system can promote human cooperation and be more efficient than peer punishment due to its ability to overcome coordination failure and free-riding problems [38–40].

This paper engages in discussions about peer punishment and centralized sanctioning by developing a formal iterative two-level trust game with incomplete information. More specifically, it proposes a hierarchical structure which not only simultaneously includes individual trust and institutional trust, but also examines how centralized sanctioning might affect reciprocity between the trustor and the trustee. In each period of the newly proposed game, an authority who moves after the trustee is added. If the trustee honoured trust, then the focal period of the game ends; if the trustee abused trust, then a choice is granted to the authority, who can either impose a costly punishment on the defective trustee or not punish the defection. Meanwhile, information is incomplete in the sense that actors are not fully informed on other actors’ utility functions and preferences. Note the hierarchical structure and the role of the authority in this paper differ from those in previous literature on altruistic third-party punishment and human cooperation [41–49]. In our model, the authority engages in repeated interactions rather than a one-shot game. In addition, the authority’s total utilities are dependent on his own and other actors’ behaviour. Thus he is considered a strategic purposive actor rather than an altruist who gains no economic benefits from costly punishment. Therefore, the configurations of this model do not intend to examine human altruism; instead, we focus on how centralized sanctioning institutions and incomplete information may affect reciprocity at the individual level. This extension could improve the applicability of the two-level trust game in more complex settings. By linking equilibrium strategies of the traditional trust game with those of the newly proposed model, one could compare the effects of centralized sanctioning and peer punishment on trust and reciprocity.

This paper is organized as follows. First, a two-level trust game which is built upon the traditional baseline trust game is introduced. Then, the sequential equilibrium is formally derived for the two-level trust game under a scenario in which information is incomplete about both the trustee and the authority. Finally, the paper concludes with theoretical and empirical implications of the two-level trust game.

## Method

### The baseline trust game with incomplete information

**Fig 2:**
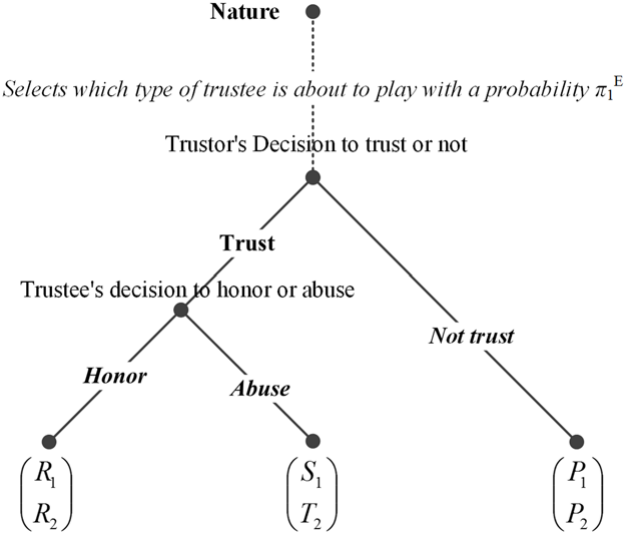
Extensive form of a baseline trust game with incomplete information.

The formal game theoretic analysis begins with a review of a baseline model which is built upon the trust game presented in Fig 1 [7,10,50,51]. The baseline model includes two important features. The first one is a move by nature, before the game starts, deciding which type of trustee will participate in the game (see Fig 2). This entails that it is assumed two types of trustees exist in nature—the G-type (good) and the B-type (bad). Both types of trustees are utility maximisers. Yet they have different preferences. The G-type trustees have stronger altruistic tendencies and therefore always feel more satisfied by honouring trust than abusing trust. On the contrary, the B-type trustees have stronger selfish tendencies and therefore prefer abusing trust than honouring trust in a one-shot game. This is a plausible assumption as it reflects the coexistence of opportunists and altruists in empirical settings [52–54]. The trustee knows his type, yet information is incomplete in the sense that, at the beginning of the game, the trustor does not know which type of trustee will be his counterpart. Let *π*_1_^E^ be the probability that the trustor assigns at the beginning of the game to the event that the trustee is a G-type.

The second feature is a continuation of the game. The game is finitely repeated without the assumption of discounting utilities. Hence the total utility any actor receives during the game is the total undiscounted sum of utility that he obtains in each game period. Moreover, anyone in this game knows exactly how many periods will last in the game.

Under circumstances of complete information, backward induction informs us that no trust should be placed in finitely repeated trust games if the trustor knew he would encounter a B-type trustee; however, with slight uncertainties about the type of the trustee, the outcome of the game would have been substantially changed in a way that even B-type trustees might honour trust in early periods of the game. The sequential equilibrium of the baseline trust game with incomplete information consists of three phases; namely, stable trust, randomization and no trust [51,55]. Bower, Garber, and Watson (1996) provided a comprehensive proof of this sequential equilibrium when the baseline trust game is played twice. To avoid complexity and ensure consistency in the following analysis, the results for the two-period baseline game are summarised in Table 1, where the notations are defined as follows:

*w*_1_ & *w*_2_ = probabilities that the trustor *A*_1_ places trust at period I & II.

*r*_1_ & *r*_2_ = probabilities that the B-type trustee *A*_2_ honours trust at period I & II.

**Table 1:** Sequential equilibria for the baseline trust game.

Case	Equilibrium strategies
1. $\pi_{1}^{E}>\frac{S_{1}}{R_{1}-S_{1}}$	*w*_1_ = *r*_1_ = *w*_2_ = *r*_2_ = 1
2. $\pi_{1}^{E}=\frac{S_{1}}{R_{1}-S_{1}}$	*w*_1_ = *r*_1_ = 1; *w*_2_≥ (*T*_*2*_—*R*_*2*_)/*T*_*2*_
3. ${\left(\frac{S_{1}}{R_{1}-S_{1}}\right)}^{2}<\pi_{1}^{E}<\frac{S_{1}}{R_{1}-S_{1}}$	*$w_{1}=1;\;r_{1}=-\pi_{1}^{E}/S_{1}(1-\pi_{1}^{E});\;w_{2}=\left(T_{2}-R_{2}\right)/T_{2}$*
4. $\pi_{1}^{E}={\left(\frac{S_{1}}{R_{1}-S_{1}}\right)}^{2}$	*$w_{1}=1;\;r_{1}=-\pi_{1}^{E}/S_{1}(1-\pi_{1}^{E});\;w_{2}\leq \left(T_{2}-R_{2}\right)/T_{2}$*
5. $\pi_{1}^{E}<{\left(\frac{S_{1}}{R_{1}-S_{1}}\right)}^{2}$	*w*_1_ = *w*_2_ = 0

Source: adapted from (Bower, et al., 1996). For analytical simplicity, it is assumed that *P*_1_ = *P*_2_ = *0* when no trust is placed, and hence *S*_1_ *<0*. *π*_1_^E^ is common knowledge.

This result exhibits that whether the trustor places trust is mainly dependent on his *ex ante* belief about the probability that the trustee is a G-type, the number of periods to be played and the RISK for him to place trust, where RISK = *S*_1_ */(S*_1_ *-R*_1_ *)* [55]. Therefore, it is easy to conclude that the first (trust) phase of the model will be longer under three conditions; namely, a higher *ex ante* probability that the trustee is a G-type, a larger number of periods to be played and a smaller risk of placing trust for the trustor.

### The two-level Trust game with incomplete information

**Fig 3:**
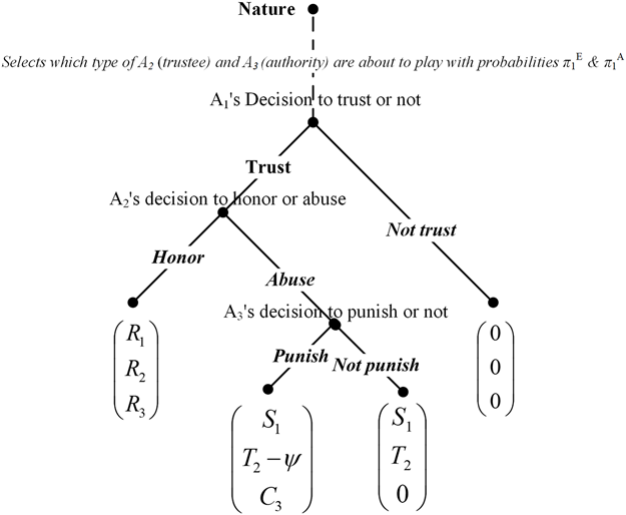
Extensive form of a two-level trust game with incomplete information.

The two-level trust game is an extension of the baseline model (see Fig 3). The term “two-level” emphasises a hierarchical structure and a newly introduced actor—the authority.

Let the trustor, the trustee and the authority be named by the order of their actions as *A*_1_ and *A*_2_ and *A*_3_. In a focal period of the two-level trust game, the authority would receive a reward *R*_3_ if trust was placed and honoured; nevertheless, if trust was placed and abused, then the authority would not receive the reward *R*_3_ and he must make a binary choice between punishing (C) and not punishing (D) the defective trustee. By punishing defective trustees, the authority *A*_3_ can impose a punishment *ψ* on them. This punishment is costly for the authority as it associates with a negative cost *C*_3_. The punishment will not change the utility of the trustor; however, it reduces the utility of the defective trustee to *T*_2_-*ψ*. It is assumed that (*T*_2_ *-ψ)<R*_2_ and hence a rational trustee would not have abused trust if he knew that he was going to be punished. In the baseline model, the type of the trustee is unknown to the trustor at the beginning of the game. Likewise, it is sensible to make a similar assumption about the type of the authority. Namely, two types of authorities are assumed to co-exist in nature—an altruistic G-type who always prefer to punish defective trustees and an opportunistic B-type who are reluctant to punish if it was only a one-shot game. It is assumed that *R*_3_ *+C*_3_ *>0* and thus the B-type *A*_3_ has an incentive to bear a short-term cost for a long-term reward in repeated interactions. It is continued to assume *P*_1_ = *P*_2_ = *P*_3_ = *0* when no trust is placed for analytical simplicity.

## Results

In this section, the sequential equilibrium strategies for all actors in the two-level trust game are derived. Let *π*_n_^E^ and *π*_n_^A^ respectively denote the probabilities assigned to the event that the trustee *A*_2_ and the authority *A*_3_ are a G-type at period *n*, where the subscript *n* denotes the period of the game. Note that *π*_n_^E^ and *π*_n_^A^ are common knowledge. For analytical simplicity, the game is assumed to be played twice (*n = 1*, *2*) and there will be no discounted utilities in the game. To derive the sequential equilibrium of the game, this paper follows the approach developed by Bower, et al. (1996). Each decision node on the game tree is indexed and worked backwards starting with the second period.

### Node 6: Period II, *A*_3_’s decision when *A*_2_ abused trust

There are two types of *A*_3_. The G-type *A*_3_ surely punishes. The B-type *A*_3_ surely defects because the game will immediately end after *A*_3_’s action and there will be no future utilities to offset the B-type *A*_3_’s costs for punishing. It is a unique equilibrium continuation.

### Node 5: Period II, *A*_2_’s decision to honour or abuse when trusted by *A*_1_

There are two types of *A*_2_. The G-type *A*_2_ always honours trust. The B-type *A*_2_ honours trust only if his expected utilities for choosing C are larger than those of choosing D. In other words, the B-type *A*_2_ honours trust when the authority is sufficiently trustworthy and the punishment on defectors is sufficiently high.

Mathematically that is,$R_{2}>\pi_{2}^{A}(T_{2}-\psi )+(1-\pi_{2}^{A})T_{2}$, and algebra yields,

$$\pi_{2}^{A}>\frac{T_{2}-R_{2}}{\psi }$$ (1)

Otherwise, the B-type *A*_2_ abuses trust. Note that the B-type *A*_2_ may randomize with probability r_2_ ∊ [0,1] when $\pi_{2}^{A}=\left(T_{2}-R_{2}\right)/\psi$.

### Node 4: Period II, *A*_1_’s decision to place trust or not

*A*_1_ only places trust when his expected utilities for placing trust are larger than zero, the latter of which is his expected utilities for not placing trust in period II. Different from the baseline model, now *A*_1_’s expected utilities not only depend on the probability that *A*_2_ is a G-type who will honour trust for sure, they also depend on the probability that *A*_3_ is a G-type who will punish the B-type *A*_2_; in the latter case, even the B-type *A*_2_ might honour trust in the last round of the game given that there is a high belief that *A*_3_ would punish *A*_2_ should he abused trust. In other words, *A*_1_ will place trust if either the probability that *A*_2_ is a G-type or the probability that *A*_3_ is a G-type is sufficiently high.

It is already established in the analysis of Node 4 that the B-type *A*_2_ honours trust on condition of Inequality (1). Thus, it is clear that *A*_1_ will place trust when *π*_2_^A^ *>(T*_2_ *-R*_2_ *)/ψ*. In addition, *A*_1_ will also place trust if he believes the trustee is sufficiently trustworthy even when Inequality (1) is not satisfied. That is to say although the B-type *A*_2_ surely defects, *A*_1_ will place trust when $\pi_{2}^{E}R_{1}+(1-\pi_{2}^{E})S_{1}>0$, and algebra yields

$$\pi_{2}^{E}>\frac{S_{1}}{S_{1}-R_{1}}$$ (2)

Note that *A*_1_ may randomize with probability *w*_2_ ∊ [0,1] when *π*_2_^E^ = *S*_1_ */(S*_1_ *-R*_1_ *)*. Here the first two-level outcome is reached; namely, the trustor *A*_1_ will place trust at period II if either Inequality (1) or (2) is satisfied.

### Node 3: Period I, *A*_3_’s decision to punish or not when *A*_2_ defected

Node 3 is only reached when the trustee *A*_2_ abused trust in period I, which suggests *A*_2_ is B-type and *π*_2_^E^ = *0*. At node 3, the G-type *A*_3_ always punishes. Note that if the B-type *A*_3_ did not punish the defective *A*_2_ in period I, then it would be revealed that *A*_3_ is B-type too, which leads to *π*_2_^A^ = *0* as well as zero utility for all actors in period II. The B-type *A*_3_ would, however, punish only if his expected utilities in period II are no smaller than his costs for punishing the defective *A*_2_ in period I.

Suppose in equilibrium a B-type *A*_3_ chooses to punish with a probability *q*_r_. Note that by Bayes’ rule,

$$\pi_{2}^{A}=\frac{\pi_{1}^{A}}{\pi_{1}^{A}+(1-\pi_{1}^{A})q_{r}}$$ (3)

Let *q*_r_* be defined by

$$\frac{T_{2}-R_{2}}{\psi }=\frac{\pi_{1}^{A}}{\pi_{1}^{A}+(1-\pi_{1}^{A})q_{r}^{*}}$$

and thus

$$q_{r}^{*}\equiv \frac{\pi_{1}^{A}(\psi +R_{2}-T_{2})}{\left(T_{2}-R_{2})(1-\pi_{1}^{A}\right)}$$ (4)

In the following it is shown by contradiction that *q*_r_* is the equilibrium value of *q*_r_.

If *q*_r_ *>q*_r_*, then *π*_2_^A^ *<(T*_2_ *-R*_2_ *)/ψ* and the B-type *A*_3_’s continuation utility in period II would be zero; because, *A*_1_ knew that *A*_2_ would have abused trust if *A*_1_ places trust in period II, therefore *A*_1_ would not place trust in the first place and everyone receives zero in the second round. In this case, the B-type *A*_3_ would strictly prefer choosing not to punish in period I, which implies *q*_r_ = 0 (a contradiction).

If *q*_r_ *<q*_r_*, then *π*_2_^A^ *>(T*_2_ *-R*_2_ *)/ψ* and the B-type *A*_3_ expects a continuation utility of *R*_3_ in period II; because *A*_1_ knew that *A*_2_ would have honoured trust if *A*_1_ places trust in the first place. Thus the trust would be placed and honoured in period II. Given it was stipulated that *R*_3_ is strictly larger than the cost for a B-type *A*_3_ to punish defective *A*_2_, the B-type *A*_3_ then strictly prefers choosing to punish, which implies *q*_r_ = 1 (a contradiction).

Furthermore, the randomization probabilities for *A*_1_ and *A*_2_ in equilibrium, when *π*_2_^E^ = *S*_1_ */(S*_1_ *-R*_1_ *)* and *π*_2_^A^ = *(T*_2_ *-R*_2_ *)/ψ*, are selected such that the actors who respectively move prior to *A*_1_ and *A*_2_ are indifferent in the periods before [55]. For *A*_2_, the trustor *A*_1_ must be indifferent between placing and not placing trust, that is, *r*_2_ *R*_1_ *+(1-r*_1_ *)S*_*1*_ = 0 and thus *r*_2_ = *S*_1_ */(S*_1_ *-R*_1_ *)*; for *A*_1_, the authority must be indifferent between imposing and not imposing punishment, that is *w*_2_ *r*_2_ *R*_3_ *+C*_*3*_ = *0*. Replacing *r*_2_ with *S*_1_ */(S*_1_ *-R*_1_ *)* and simple algebra yields *w*_2_ = *C*_3_ *(R*_1_ *-S*_1_ *)/(R*_3_ *S*_*1*_ *)*. In a nutshell, the randomization probability cannot be either too high or too low to alter the choice of the actor who moves in the period before.

### Node 2: Period I, *A*_2_’s decision to honour or abuse trust when trust is placed

The G-type *A*_2_ always cooperates. With regard to the strategy for the B-type *A*_2_, one needs to consider a two-level deduction. More specifically, the B-type *A*_2_ now simultaneously faces an internal and an external factor that influence his choice of action. The internal factor is peer punishment which might be imposed on the B-type A_2_ and reduces his potential long-term benefits. The peer punishment might drive the B-type A_2_ pretend to be a G-type. This is a well-established notion according to the baseline model and it has been tested by many laboratory experiments [12,56]. The external factor, on the other hand, is introduced by the imposition of the authority. In this two-level trust game, it is a centralized sanctioning institution and an extra constrain on possible defective behaviours of the B-type *A*_2_. Therefore, the B-type *A*_2_ has another motivation to cooperate in addition to his concern for peer punishment.

The centralized sanctioning is reflected by *A*_2_’s expected utilities. He will honour trust if his expected utilities for choosing C are no smaller than choosing D at period I. That is,

$$R_{2}>(T_{2}-\psi )\pi_{1}^{A}+(1-\pi_{1}^{A})q_{r}^{*}(T_{2}-\psi )+(1-\pi_{1}^{A})(1-q_{r}^{*})T_{2}$$

replace *q*_r_* with Eq (4) and algebra yields,

$$\pi_{1}^{A}>{\left(\frac{T_{2}-R_{2}}{\psi }\right)}^{2}$$ (5)

When Inequality (5) cannot be satisfied, the scenario can be simply viewed as a baseline model in which the authority does not exist. It basically suggests that the authority is so untrustworthy to such extent that *π*_1_^*A*^ *<[(T*_2_ *-R*_2_ *)/ψ]* ^2^. In this case, the B-type *A*_2_ may randomize with probability r_1_ ∊ [0,1]in equilibrium as proved by Buskens [55] and Bower et al. [51]. The process of randomization probability selection is similar to what has been shown in the analysis of Node 3. Without further duplication, the randomization probability is,

$$r_{1}=\frac{\pi_{1}^{E}R_{1}}{(\pi_{1}^{E}-1)S_{1}}$$ (6)

### Node 1: Period I, *A*_1_’s decision to place trust or not

Similar to the analysis of Node 4, *A*_1_ chooses to place trust under two circumstances. One is that the B-type trustee honours trust given their anticipation that there is a sufficiently high probability the authority will punish. This condition is illustrated by the analysis of Node 2 and Inequality (5). When Inequality (5) cannot be satisfied, or to put it another way, when the authority’s trustworthiness does not reach an adequate level, an alternative condition for the trust placement is that the probability that the trustee is a G-type is sufficiently high. This is clearly true when *π*_1_^*E*^ *>S*_1_ */(S*_1_ *-R*_1_ *)*. If *π*_1_^*E*^≤*S*_1_ */(S*_1_ *-R*_1_ *)*, then it implies

$$\pi_{1}^{E}R_{1}+(1-\pi_{1}^{E})r_{1}R_{1}+(1-\pi_{1}^{E})(1-r_{1})S_{1}>0$$

Substitution of *r*_1_ according to Eq (6) and algebra yields

$$\pi_{1}^{E}<{\left(\frac{S_{1}}{R_{1}-S_{1}}\right)}^{2}$$ (7)

Therefore, *A*_1_ places trust with certainty at period I when either Inequality (5) or Inequality (7) is satisfied. There will be no trust placement if neither inequality can be satisfied.

To sum up, the sequential equilibrium of the two-level trust game inherits characteristics of the baseline model. In particular, *π*_1_^E^ (Actors’ *ex ante* belief in the probability that the trustee *A*_2_ is a G-type) and RISK still constitute important factors for trust. In addition, new features have been developed. *π*_1_^A^ (Actors’ *ex ante* belief in the probability that the authority *A*_3_ is a G-type) and an alternative version of temptation, TEMPP = *(T*_2_ *-R*_2_ *)/ψ* (recall that TEMP = *(T*_2_ *-R*_2_ *)/T*_2_), are incorporated into the equilibrium. It should be noted that the *ex ante* beliefs in the probability that *A*_2_ and *A*_3_ is a G-type respectively fall into five categories as illustrated in Table 2. These five categories are labelled as Full optimism (FO), High optimism (HO), Intermediate optimism (IO), High pessimism (HP) and Full pessimism (FP) with a descending degree of confidence in *A*_2_ and *A*_3_ being a G-type.

**Table 2:** Categories for the two-level trust game with incomplete information about both the authority and the trustee.

Category I: Belief that A_3_ is a G-type	Category II: Belief that A_2_ is a G-type	Description
*$\pi_{1}^{A}>\frac{T_{2}-R_{2}}{\psi }$*	$\pi_{1}^{E}>\frac{S_{1}}{R_{1}-S_{1}}$	Full optimism (FO)
*$\pi_{1}^{A}=\frac{T_{2}-R_{2}}{\psi }$*	$\pi_{1}^{E}=\frac{S_{1}}{R_{1}-S_{1}}$	High optimism (HO)
*${\left(\frac{T_{2}-R_{2}}{\psi }\right)}^{2}<\pi_{1}^{A}<\frac{T_{2}-R_{2}}{\psi }$*	${\left(\frac{S_{1}}{R_{1}-S_{1}}\right)}^{2}<\pi_{1}^{E}<\frac{S_{1}}{R_{1}-S_{1}}$	Intermediate optimism (IO)
*$\pi_{1}^{A}={\left(\frac{T_{2}-R_{2}}{\psi }\right)}^{2}$*	$\pi_{1}^{E}={\left(\frac{S_{1}}{R_{1}-S_{1}}\right)}^{2}$	High pessimism (HP)
*$\pi_{1}^{A}<{\left(\frac{T_{2}-R_{2}}{\psi }\right)}^{2}$*	$\pi_{1}^{E}<{\left(\frac{S_{1}}{R_{1}-S_{1}}\right)}^{2}$	Full pessimism (FP)

The incomplete information about both the trustee and the authority reconstructs the baseline trust game. The result of the newly proposed two-level trust game suggests that the equilibrium strategies for both the trustor and B-type actors are altered. There are 25 cases which involve different combinations of *ex ante* beliefs in the types of the trustee and the authority. The equilibrium strategies for the trustor, the B-type trustee and the B-type authority in each scenario are summarized in Table 3.

**Table 3:** Sequential equilibrium strategies for the two-level Trust game.

Case	Belief in the authority	Belief in the trustee	Equilibrium Strategies
*I-V*	*π*_*1*_^*A*^ = *FO*	*π*_*1*_^*E*^ = *FO*, *HO*, *IO*, *HP*, *FP*	*w*_1_ = *r*_1_ = *w*_2_ = *r*_2_ = *1*;
*VI-X*	*π*_*1*_^*A*^ = *HO*	*π*_*1*_^*E*^ = *FO*	*w*_1_ = *r*_1_ = *w*_2_ = *1; r*_2_ = *S*_1_ */(S*_1_ *-R*_1_ *)*
		*π*_*1*_^*E*^ = *HO*, *IO*, *HP*, *FP*	*w*_1_ = *r*_1_ = *1; w*_2_ *≥ C*_3_ *(R*_1_ *-S*_1_ *)/(R*_3_ *S*_1_ *); r*_2_ = *S*_1_ */(S*_1_ *-R*_1_ *)*
*XI-XV*	*π*_*1*_^*A*^ = *IO*	*π*_*1*_^*E*^ = *FO*	*w*_1_ = *r*_1_ = *w*_2_ = *1; r*_2_ = *0*
		*π*_*1*_^*E*^ = *HO*	*w*_1_ = *r*_1_ = *1; w*_2_ *≥C*_3_ *(R*_1_ *-S*_1_ *)/(R*_3_ *S*_1_ *); r*_2_ = *0*
		*π*_*1*_^*E*^ = *IO*, *HP*, *FP*	*w*_1_ = *r*_1_ = *1; w*_2_ = *C*_3_ *(R*_1_ *-S*_1_ *)/(R*_3_ *S*_1_ *); r*_2_ = *0*
*XVI-XX*	*π*_*1*_^*A*^ = *HP*	*π*_*1*_^*E*^ = *FO*	*w*_1_ = *r*_1_ = *w*_2_ = *1; r*_2_ = *0*
		*π*_*1*_^*E*^ = *HO*	*w*_1_ = *r*_1_ = *1; w*_2_ *≥C*_3_ *(R*_1_ *-S*_1_ *)/(R*_3_ *S*_1_ *); r*_2_ = *0*
		*π*_*1*_^*E*^ = *IO*, *HP*, *FP*	*w*_1_ = *1; r*_1_ = *π*_1_^*E*^ *R*_1_ */(S*_1_ *-π*_1_^*E*^ *S*_1_ *); q*_r_ = *π*_1_^*A*^ *(ψ+R*_2_ *-T*_2_ *)/[(T*_2_ *- R*_2_ *)(1-π*_1_^*A*^ *)]; w*_2_ = *C*_3_ *(R*_1_ *-S*_1_ *)/(R*_3_ *S*_1_ *); r*_2_ = *0*
*XXI-XXV*	*π*_*1*_^*A*^ = *FP*	*π*_*1*_^*E*^ = *FO*	*w*_1_ = *r*_1_ = *w*_2_ = *1; r*_2_ = *0*
		*π*_*1*_^*E*^ = *HO*	*w*_1_ = *r*_1_ = *1; w*_2_ *≥C*_3_ *(R*_1_ *-S*_1_ *)/(R*_3_ *S*_1_ *); r*_2_ = *0*
		*π*_*1*_^*E*^ = *IO*, *HP*	*w*_1_ = *1; r*_1_ = *π*_1_^*E*^ *R*_1_ */(S*_1_ *-π*_1_^*E*^ *S*_1_ *); q*_r_ = *π*_1_^*A*^ *(ψ+R*_2_ *-T*_2_ *)/[(T*_2_ *- R*_2_ *)(1-π*_1_^*A*^ *)]; w*_2_ = *C*_3_ *(R*_1_ *-S*_1_ *)/(R*_3_ *S*_1_ *); r*_2_ = *0*
		*π*_*1*_^*E*^ = *FP*	*w*_1_ = *w*_2_ = *0*

#### Case I-V

When *π*_1_^A^ = *FO*, the actors are initially very optimistic about the authority’s type. In this case, maximum trust and reciprocity can be achieved regardless of actors’ *ex ante* beliefs in the trustee. The sequential equilibrium only includes pure strategies in the sense that the trustor places trust and the trust is honoured in both periods. Even the B-type trustee honours trust in the last period of the game. The authority enjoys the reward for the establishment of trust without entering the game. These cases yield the highest group utilities for all actors.

#### Case VI-X

When *π*_1_^A^ = *HO*, the actors are still optimistic about the authority’s type, but their equilibrium strategies might vary. The trustor will place trust in both periods if *π*_1_^E^ = *FO* given it indicates his full confidence in the trustee. He will otherwise place trust in period I and randomize with a high probability in period II regardless of his *ex ante* beliefs in the trustee. On the other hand, the B-type trustee will always honour trust in Period I and always randomize in period II. His randomization probability is determined by a polynomial which represents the risk for the trustor to place trust.

#### Case VI-X

When *π*_1_^A^ = *IO*, it implies an intermediate degree of optimism about the authority’s type. The trustor will place trust in both periods if he is fully confident in the trustee (*π*_1_^E^ = *FO*) and he will randomize in period II otherwise. His randomization probability is determined by a polynomial consists of various parameters such as his risk for placing trust, the costs *C*_3_ for the authority to impose punishment and the rewards *R*_3_ for the authority if trust is placed and honoured. The B-type trustee will surely cooperate in period I and always defect in period II considering the *ex ante* belief in the authority’s type is not sufficiently high.

#### Case XVI-XX

When *π*_1_^A^ = *HP*, the actors are generally pessimistic about the authority’s type. However, trust can be still placed and honoured in period I if *π*_1_^E^ is relatively high; because, the B-type trustee has incentives to pretend to be a G-type in order to receive higher long-term benefits. If the degree of optimism about the trustee’s type is intermediate or relatively low, the B-type trustee will randomize in Period I and always abuse trust in Period II. The B-type authority will also randomize if the trustee abused in Period I. The trustor may also place trust with a probability in Period II. There will be no trust until the trustor stops placing trust or trust is abused and defection is unpunished.

#### Case XXI-XXV

When *π*_1_^A^ = *FP*, it implies that the actors are fully pessimistic about the authority’s type. The game then turns into a scenario similar to the baseline game in the sense that the authority barely has any impact over the actors’ choices of action. Basically, the sequential equilibrium strategies for the trustor and the B-type trustee are similar to what is presented in Table 1.

## Discussion and Conclusion

This paper has presented a two-level trust game with incomplete information. This two-level configuration introduces several new features and it depicts a hierarchical structure of interactions in which the effects of centralized sanctioning on trust and reciprocity can be examined. Many new insights have been developed from the game theoretic analysis of the two-level trust game.

Firstly, the sequential equilibrium of our model simultaneously includes both the risk for placing trust (RISK) and the temptation for abusing trust (TEMPP) as key factors for cooperation at the individual level. This is an important advancement because traditional formal models only include one of them as a key factor for cooperation in social dilemmas. For instance, the Perfect Folk Theorem explains cooperation with indefinitely repeated games. It suggests that rational actors might cooperate in social dilemmas as long as the discounting factor *β* is sufficiently large when compared to TEMP. The baseline trust game explains cooperation with incomplete information. As shown in Table 1, RISK plays a much more important role than TEMP in determining the length of cooperation period in the baseline trust game. Although these two mechanisms (indefinitely repetition and incomplete information) are well-known for bringing about cooperation in social dilemmas, no previous formal models have simultaneously incorporated TEMP and RISK into critical conditions for cooperation. The proposed two-level model, however, reaches such an integrated result as indicated by Inequalities 5 and 7.

Secondly, the two-level trust game provides an opportunity to compare its equilibrium strategies with those of the baseline model in which no overarching authority participates. This comparison leads to an interesting postulation suggesting that even a slight uncertainty about the authority’s type might significantly increase the level of trust and reciprocity at the individual level. In previous laboratory experiments, scholars often observe a decrease of trustworthiness in the last few rounds of the baseline trust game. This phenomenon is referred as the end-game effect [29]. Yet in some cases of the two-level trust game, full trustfulness and full trustworthiness can be obtained such that the end-game effect can be alleviated. This is mainly due to two reasons. One is that the conditions for trust placement is relaxed—a high trustworthiness of either the trustee or the authority is sufficient for the trustor to place trust. The other reason is that the condition for the B-type trustee to honour trust is relaxed. Specifically, the imposition of the authority creates an additional incentive for the B-type trustee to pretend to be a G-type since he is afraid to be punished when the *ex ante* belief *π*_1_^A^ is sufficiently high. Therefore, the incomplete information with regard to the type of the authority reshapes each actor’s equilibrium strategies. It produces favourably impacts on conditions for trust and reciprocity.

Lastly, the two-level set up of the trust game brings new factors, including *ψ*, *R*_3_ and *C*_3_, into the baseline model. They all have different effects on the sequential equilibrium. The amount of centralized sanction imposed on defective trustees plays a key role in determining equilibrium strategies for both the trustor and the trustee. When the *ex ante* beliefs are common knowledge and fixed, the harsher the sanction is the more likely trust is placed and honoured. Yet the utilities associated with the authority’s reward *R*_3_ and costs *C*_3_ only play a peripheral role of determining the randomization period for the trustor.

Despite the above theoretical interest in the effects of centralized sanctioning and incomplete information on human cooperation, empirical evidence testing these theories is limited. Pluralistic methods, including experimental approaches, are needed to develop more comprehensive, accurate and well-specified explanations of these game theoretic postulations [57,58]. Testable hypotheses could be generated from the formal two-level trust game and examined in experiments in future research. For instance, Buskens et al. [29,55,59] and Anderhub et al. [30] have analysed, in experimental settings, the sequential equilibrium of the baseline trust game with two or three actors [6,51,60]. An important finding from these studies is the end-game effect. The analyses of our two-level trust game, however, suggests that the end-game effect may be weakened by the imposition of a centralized sanctioning institution and incomplete information. This leads to the following hypotheses: 1) In the condition of the two-level trust game, compared to the condition of the baseline trust game, the likelihood of trustworthiness of the trustor and trustworthiness of the trustee is higher; 2) The likelihood of trustfulness and trustworthiness decrease slower in the last few rounds of the two-level trust game than that in the baseline trust game. By moderate modifications to previous experimental configurations by Buskens et al. and Anderhub et al., one could test these hypotheses and compare the effects of centralized sanctioning on trust and reciprocity in alternative lab experiments.

To put the two-level model in a broader context, it could shed some light on two interrelated types of trust; namely, individual trust between the trustor and trustee as well as institutional trust between the individuals and the authority. The result of the two-level game demonstrates that the imposition of an authority produces a synergetic effect on trust and reciprocity. This result, to some extent, echoes with extensive arguments concerning the relationship between “trust in the state” and “social trust” [14,61–63]. Future research could further explore such conjectures with more empirical evidence collected in the field.
